# Surgery

**Published:** 1909-09

**Authors:** Harold F. Mole


### SURGERY.

The question of when and how to operate in cases of **exophthalmic goitre** is still in this country, I believe, unsettled, and judged by the diverse views expressed by those in other countries the same condition holds in them, though they appear to operate much more freely than we do. Now that the disease is definitely known to be due to an excessive secretion by, and an excessive absorption from, the thyroid gland, it seems only rational that the treatment should be directed towards lessening the secretion in some way or to the removal of part of the secreting substance. By the way, the name usually given to the disease abroad, viz. hyperthyroidism or thyrotoxicosis (Kocher), is much better, and more accurate and expressive than the terms usually used. The mortality from operation used to be so high that, I believe, many surgeons in this country still refuse to operate. Long series of cases have, however, been recently reported from foreign clinics with very satisfactory results. Dunhill[1] shows only one death in eighty-eight cases, and the brothers Mayo[2] one death in a recent series of seventy-cases, but a mortality rate of 5 per cent. in a series of two hundred cases. There is a very great difference in the results according as the case is one of primary Graves's disease, or the symptoms peculiar to the latter are engrafted on a simple goitre of some years standing. In the latter case the mortality is but little higher than for operation in cases of simple goitre. There seems to be a great difficulty in deciding which cases should be operated on and which not. No rules can be laid down. Most are agreed that medical treatment should have a good trial first, and if the case is not improving, and especially if the case is getting worse, then operation should be performed. Ferguson,[3] however, says that until some better treatment is discovered he will operate on every case he comes across.

When patients die after operation in Graves's disease, they do so from acute thyroidism, that is to say an acute increase in all the symptoms present with high temperature and often albuminuria. It was supposed to be due to the setting free of thyroid secretion from the cut or torn surface of the gland and its absorption from the wound. That this is not a sufficient explanation in all cases is shown by similar symptoms arising after operations on other parts of the body in these cases, and also in some cases under excitement when no operation has been performed. Assuming that any given case is an appropriate one for operation, there are the following proceedings available: Removal of portions of the cervical sympathetic ligation of thyroid arteries, and removal of portions of the gland itself. With regard to the first, some satisfactory results have been given by Curtis,[4] but some of the cases relapsed. The operation is more difficult than the others, and the mortality is equally high, and so it is no longer practised. In those cases which benefited, the assumption is that the secretion was lessened by removing the sympathetic nerve-supply to the gland. The nerve trunk, with its superior and middle cervical ganglia, were removed on both sides. With regard to the second procedure, viz. ligation of the thyroid

[1] *Brit. M. J.*, 1909, i, 1222, [2] *Tr. Am. Surg. Ass.*, 1908, xxvi, 397.

[3] *Surg., Gynec., and Obst.*, 1909, viii, 279.

[4] *Ann. Surg.*, 1903, xxxviii, 161; 1906, xliii, 335.

arteries, this is only a palliative measure, and can be done as a preliminary to later removal of the gland when it is considered that the patient is too ill to stand the latter. It amounts to this, then, that in any case in which an operation is considered advisable the best thing to do is to remove a portion of the gland. Before considering this more in detail it will be well to pay attention to the preliminary treatment of the patient and the question of the anæsthetic, about which there is still much difference of opinion.

Crile[1] has called attention to the psychic factor in Graves's disease, on which he lays the very greatest stress. He takes it for granted that if a sufficient amount of the thyroid gland be successfully removed cure will follow ; if only partial relief, then enough has not been removed. He considers that many patients get nervous and excited before operation with exaggeration of their symptoms, which continues through the operation up to their death afterwards. He was fortunate in coming across two dogs with Graves's disease on which to make observations. When the dogs were excited by fear or anger there was, about six hours later, increased temperature and tachycardia, tremor, etc., and often gastro-intestinal symptoms. If the dog was excited and then anæsthetised marked hyperthyroidism followed, but if after three days' rest and the preliminary administration of morphine the anæsthetic was administered for the same length of time and in the same way, then no hyperthyroidism followed. From these and other laboratory experiments the following hypothesis was formed : "That in Graves's disease the most powerful factor producing hyperthyroidism is psychic excitation ; that in some way, either directly or indirectly, psychic excitation discharges into the circulation an excessive amount of thyroid secretion, which in itself may cause death ; that the greatest factor in the mortality is not the operation *per se* if well done, but what has occurred before the operation. In other words, at the time the surgeon makes his first incision the fate of the patient has been already sealed." He endeavours, therefore, to so lead up to his operation that the patient shall be unaware of it, or indeed that she is taking an anæsthetic, and so thus avoid all psychic disturbances. In addition to rest in bed, diet, etc., the trained anæsthetist every morning goes through the complete form of giving the anæsthetic, solutions of volatile oils being dropped on the ether mask, and this being continued and explained as part of the treatment until it is considered that an appropriate time has been reached for operating. Bromides are then administered on the night preceding, and a hypodermic of morphia in the morning of operation ; the room is darkened, and the anæsthetist commences with the volatile oils he has been previously using, saying it will be rather stronger as he gradually adds the ether, and in this way the operation is done without the patient's knowledge, and the gland

[1] *Ann. Surg.*, 1908, xlvii, 864 ; *Tr. Am. Surg. Ass.*, 1908, xxvi, 391.

is thus " stolen " away, to use Crile's expression. Of course the patient knows there is going to be an operation, but does not know when. Without this special preliminary treatment other surgeons recommend rest in bed, quiet, darkened room, alcohol sponge baths, ice bag over heart if necessary, and sedatives, codeine or morphine before operation to diminish as far as possible the nervous symptoms. With regard to the choice of anæsthetic, some, Dunhill,[1] I believe, Kocher and Lewis,[2] are very strongly in favour of local anæsthesia, but, as pointed out by Ochsner[3] and others, the exceeding nervous state of the patient will militate against this method being applicable in a fair number of cases, as has been shewn when an attempt has been made and failed, and a general anæsthetic had to be administered. The advice of Lewis, that the nervousness of the patient may be overcome by the administration of a dose of whiskey before the anæsthesia, does not point strongly in its favour. Ochsner recommends ether by the open method, raising up the head when the patient is under.

Now we have to consider the operation itself. A transverse incision is made across the neck, and the gland exposed in the usual way. The question then is how much of the gland to remove. Henrick says that those surgeons who have removed most of the gland short of its entirety have obtained the best results both as to number and completeness of recoveries. Mayo removes one lobe and the isthmus. If the symptoms are not completely relieved by this, at a later period—two or three months—a portion of the remaining lobe can be removed with much less risk than at the first operation. Ferguson, in a paper previously referred to, recommends the following technique : After the gland has been thoroughly exposed and the capsule opened, the finger is passed down alongside until a free space is found about the centre and midway between the superior and inferior thyroid arteries. A stout, blunt pedicle needle is entered here inside the capsule and not too near the trachea, and passed through the parenchyma of the gland inwards and upwards to include the superior thyroid, and afterwards inwards and downwards to catch the inferior thyroid vessels. It is threaded with stout catgut, which is drawn through and tied, the same being done on each side. The gland is then cut away beyond the ligatures, only a small portion being left included in each. Any vessels that require it are tied. This procedure safeguards the parathyroids, and there is a minimum amount of hæmorrhage and shock. If the thyroid vessels are tied too near their origin, the blood supply will be cut off from the parathyroids and tetany will result. All the necessary sutures are put in, but only two or three tied, the wound being left open for two or three days for perfect drainage. All are agreed that there should be as little handling

[1] *Loc. cit.* [2] *Surg., Gynec. and Obst.*, 1909, viii, 306.
[3] *Ibid.*, p. 304.

of the gland as possible during the operation, and that very free drainage should be provided for. This is, of course, on the assumption that secretion set free from the gland during operation may be absorbed from the wound. As regards after-treatment a quart of saline should be given by rectum immediately after operation, and again, if necessary, in a few hours (Mayo[1]). If intestinal relaxation, this must be given subcutaneously. Morphia should be given for restlessness and cold to the pericardial region if marked palpitation. As soon as she is round from the anæsthetic the patient should be sat up, and get up as soon as possible afterwards.

To sum up, I would suggest the following conclusions :—

(1) That all severe cases of Graves's disease not improving under proper medical treatment, including rest in bed, should, after a period not exceeding three months (of this treatment), be submitted to operation. (2) That previous to operation every endeavour should be made to diminish the nervous excitability of the patient. (3) That the anæsthetic should, as a rule, be ether by the open method, preceded by an injection of morphia and atropin, but that in those cases where the nervous symptoms are but very slightly marked local anæsthesia should be considered. (4) That the operation should be partial thyroidectomy, removing as much of the gland as appears safe at the time ; that the gland should be submitted to as little handling as possible during the operation, and free drainage provided for afterwards.

HAROLD F. MOLE.

